# SG-APSIC1026: COVID-19 vaccine acceptance and hesitancy among primary healthcare workers in Singapore

**DOI:** 10.1017/ash.2023.10

**Published:** 2023-03-16

**Authors:** Sky Wei Chee Koh, Liow Yiyang, Victor Loh Weng Keong, Liew Seaw Jia, Chan Yiong-Huak, Doris Young

**Affiliations:** National University Polyclinics, Singapore; Singapore National University Polyclinics, Singapore; Singapore, Yong Loo Lin School of Medicine, Singapore; Singapore, Yong Loo Lin School of Medicine, Singapore; Singapore, Yong Loo Lin School of Medicine, Singapore; Singapore, Yong Loo Lin School of Medicine, Singapore

## Abstract

**Objectives:** Factors affecting COVID-19 vaccine acceptance and hesitancy among primary-care healthcare workers (HCWs) remain poorly understood. We sought to identify factors associated with vaccine acceptance and hesitancy among HCWs. **Methods:** A multicenter online cross-sectional survey was performed across 6 primary-care clinics from May to June 2021, after completion of the vaccination rollout. The following data were collected: demographics, profession, years working in healthcare, residential status, presence of chronic medical conditions, self-perceived risk of acquiring COVID-19, and previous influenza vaccination. HCWs who accepted the vaccine were asked to rank their 5 best reasons for vaccine acceptance. HCWs who were vaccine hesitant completed the 5C scale on psychological antecedents of vaccination. **Results:** Of 1,182 eligible HCWs, 557 responded (response rate, 47.1%) and 29 were excluded due to contraindications. Among 557 respondents, the vaccine acceptance rate was 94.9% (n = 501) and 5.1% were hesitant (n = 27). COVID-19 vaccine acceptance was not associated with sex, age, ethnicity, profession, number of years in healthcare, living status, presence of chronic diseases, self-perceived risk, or previous influenza vaccination. The 3 most common reasons for COVID-19 vaccine acceptance as ranked by 501 HCWs were (1) to protect their family and friends, (2) protect themselves from COVID-19, and (3) the high risk of acquiring COVID-19 because of their jobs. The 15-item questionnaire from the 5C psychological antecedents of vaccination was completed by 27 vaccine hesitant HCWs. The mean scores for the components of the 5Cs were ‘confidence’ (3.96), ‘complacency’ (3.23), ‘constraint’ (2.85), ‘calculation’ (5.79) and ‘collective responsibility’ (4.12). **Conclusions:** COVID-19 vaccine hesitancy is a minute issue among primary-care HCWs in Singapore, where the acceptance rate is 95% with a 5% hesitancy rate. Future studies can focus on other settings with higher hesitancy rates and acceptance of booster vaccinations with the emergence of the SARS-CoV-2 δ (delta) variant. Trial Registration: This study was approved by the National Healthcare Group (NHG) Domain Specific Review Board (DSRB), Singapore on April 26, 2021 (Reg No. 2021/00213).

